# Acceleration of discrete stochastic biochemical simulation using GPGPU

**DOI:** 10.3389/fphys.2015.00042

**Published:** 2015-02-13

**Authors:** Kei Sumiyoshi, Kazuki Hirata, Noriko Hiroi, Akira Funahashi

**Affiliations:** Systems Biology Laboratory, Department of Biosciences and Informatics, Keio UniversityYokohama, Japan

**Keywords:** GPGPU, stochastic simulation algorithm, direct method, parallel processing, CUDA, SBML

## Abstract

For systems made up of a small number of molecules, such as a biochemical network in a single cell, a simulation requires a stochastic approach, instead of a deterministic approach. The stochastic simulation algorithm (SSA) simulates the stochastic behavior of a spatially homogeneous system. Since stochastic approaches produce different results each time they are used, multiple runs are required in order to obtain statistical results; this results in a large computational cost. We have implemented a parallel method for using SSA to simulate a stochastic model; the method uses a graphics processing unit (GPU), which enables multiple realizations at the same time, and thus reduces the computational time and cost. During the simulation, for the purpose of analysis, each time course is recorded at each time step. A straightforward implementation of this method on a GPU is about 16 times faster than a sequential simulation on a CPU with hybrid parallelization; each of the multiple simulations is run simultaneously, and the computational tasks within each simulation are parallelized. We also implemented an improvement to the memory access and reduced the memory footprint, in order to optimize the computations on the GPU. We also implemented an asynchronous data transfer scheme to accelerate the time course recording function. To analyze the acceleration of our implementation on various sizes of model, we performed SSA simulations on different model sizes and compared these computation times to those for sequential simulations with a CPU. When used with the improved time course recording function, our method was shown to accelerate the SSA simulation by a factor of up to 130.

## 1. Introduction

Understanding biological phenomena as systems is one of the most crucial objectives in systems biology (Kitano, 2002). Mathematical modeling of biological systems and the simulation of such models will play an important role in helping us to understand unknown phenomena as systems. In systems biology, a deterministic approach, such as using ordinary differential equations (ODEs), is often used to understand the behavior of biochemical systems. A deterministic approach describes the system using molecular concentrations, and the results are the same for every realization. However, when we want to understand a system that contains a small number of molecules, such as a biochemical network in a single cell, a simulation must be executed using a stochastic approach, instead of a deterministic approach (McAdams and Arkin, 1997; Arkin et al., 1998).

The stochastic simulation algorithm (SSA) simulates the stochastic behavior of a spatially homogeneous system (Gillespie, 1977). Since stochastic approaches produce different results each time they are used, multiple runs are required in order to obtain statistical results, thus causing a large computational cost.

To reduce this large computational cost, we have focused on accelerating the SSA by using general-purpose computations on a graphics processing unit (GPGPU; Owens et al., 2007; Nvidia, 2014). GPGPU is a technology that uses a graphics processing unit (GPU) to perform numerical calculations other than those for computer graphics, its original design purpose. GPUs contain a large number of arithmetic units in order to parallelize an enormous number of simple calculations. By efficiently parallelizing a problem and simultaneously performing the calculations on these arithmetic units, we can obtain significant improvement in the performance. GPUs are now widespread; they are included in personal computers (and even in laptop computers). Because of this, the ability to harness the computing power of GPUs has rapidly developed.

We have implemented a parallel method for using SSA to simulate a stochastic model; the method efficiently utilizes a GPU, and this enables multiple realizations on the same time sequence. Thus, multiple results are obtained simultaneously, and this reduces the computational time and cost. During the simulation, for the purpose of analysis, each time course is recorded at each time step. There are some existing studies of methods used to accelerate the SSA using the GPGPU; these include (Li and Petzold, 2010) on the direct method and (Komarov and D'Souza, 2012) on the optimized direct method. These proposed methods do not provide a functionality for storing the time course data, which is essential for understanding the dynamics of a model; our implementation achieves this, and thus aids analysis.

## 2. Materials and methods

### 2.1. The SSA

The SSA was developed by Gillespie (1977), and it is an efficient and widely used algorithm for simulating the dynamics of chemically reacting systems including stochastic processes. The SSA has the following features:

- Each simulation step fires one reaction:
  During the simulation, multiple reactions do not proceed simultaneously. A single reaction is selected from the model, considering the type of reaction and its required time, and each selected reaction is executed individually.
- The reactions are selected at random:
  A reaction is selected by its propensity function. The propensity function represents its tendency to be selected; that is, a larger propensity function indicates a higher probability of being selected.
- The time required for each reaction is defined at random:
  Each reaction time τ is defined at random, but the calculated value of τ depends on the sum of the propensity function.
- Each simulation step increases or decreases the number of molecules:
  As a result of each reaction, changes are based on the number of molecules, not on their concentrations. A stoichiometry matrix is used to determine how many molecules are added or removed.

The original implementation of SSA is called the direct method. There are several additional implementations of the SSA (Gillespie, 1976; Gibson and Bruck, 2000; Cao et al., 2004; McCollum et al., 2006) that use various methods to speed up the computation time. In our implementation, we use the direct method, which is summarized as follows:

1. Initialization:
  Initialize and define the number of molecules, the reactions, and the rate constants. The reactions are specified by a stoichiometry matrix.
2. Generate uniformly distributed random numbers: *r*_1_, *r*_2_, from (0 − 1].
  These numbers determine which reaction is fired in the next step τ.
3. Calculate the propensity function *a_i_*[*i* = 0 ··· (*n* − 1)] for each reaction, where *n* is the number of reactions:
  The propensity function for each reaction will change, depending on the order of the reaction and the number of reactants. The order of each reaction should be in the range of 0th order to 2nd order; if the order of a reaction is greater than 2nd order, it should be rewritten as a combination of reactions of lower (0th–2nd) order.
4. Calculate the sum of the propensity function:
  $$a_{total}=\sum_{i\;=\;0}^{n\;-\;1}a_{i}$$
5. Calculate the reaction time: τ = (1/*a_total_*)log(1/*r*_1_).
6. Select the reaction: Select a reaction that satisfies
  $$\sum_{i\;=\;0}^{m\;-\;1}a_{i}<r_{2}\cdot a_{total}\leq \sum_{i\;=\;0}^{m}a_{i}.$$
7. Fire the selected (*m*th) reaction: Update the number of molecules, and add τ to the cumulative simulation time.
8. Termination: If the cumulative time is less than a predetermined time, return to step 2.

### 2.2. Random number generation

SSA is an algorithm that uses random numbers to represent stochastic process in a model. As shown in the previous section, the direct method uses two random numbers (*r*_1_ and *r*_2_) for each step of a simulation: one to determine which reaction is to be fired and one to determine the reaction time. The generation of these random numbers is one of the most crucial steps in SSA; it is a time-consuming task and thus impacts the total simulation time. Another concern regarding the generation of random numbers is their distribution. In SSA, a great many random numbers are generated during each simulation, so it is essential to choose a generator that can produce uniformly distributed random numbers with high dimensionality and long periodicity. In our implementation, we used the Mersenne Twister (MT), a widely used pseudorandom number generator (Matsumoto and Nishimura, 1998). We implemented a parallelized MT algorithm on a GPU; it was based on the GPGPU implementation of MT included in CUDA SDK, NVIDIA's software development kit for their parallel computing platform (Podlozhnyuk, 2007). In this implementation, the generated random numbers are stored directly in the GPU memory; this requires less communication between the host computer and the GPU.

### 2.3. Parallelization of the direct method

To accelerate the execution of the direct method, we applied both coarse-grained and fine-grained parallelization. Coarse-grained parallelization of a stochastic simulation is straightforward. In principle, a stochastic simulation requires multiple simulations using the same model and the same set of parameters, because each result shows only one possibility. To understand the dynamics and characteristics of a model, it is necessary to obtain a results from multiple simulations. Coarse-grained parallelization executes multiple simulations simultaneously. The parallelization algorithm is quite simple, in that the model is located on the global memory of a GPU, and multiple arithmetic units are engaged to execute simulations with different sets of random numbers. The acceleration of the SSA by Li and Petzold (2010) was based on coarse-grained parallelization.

**Figure 1:**
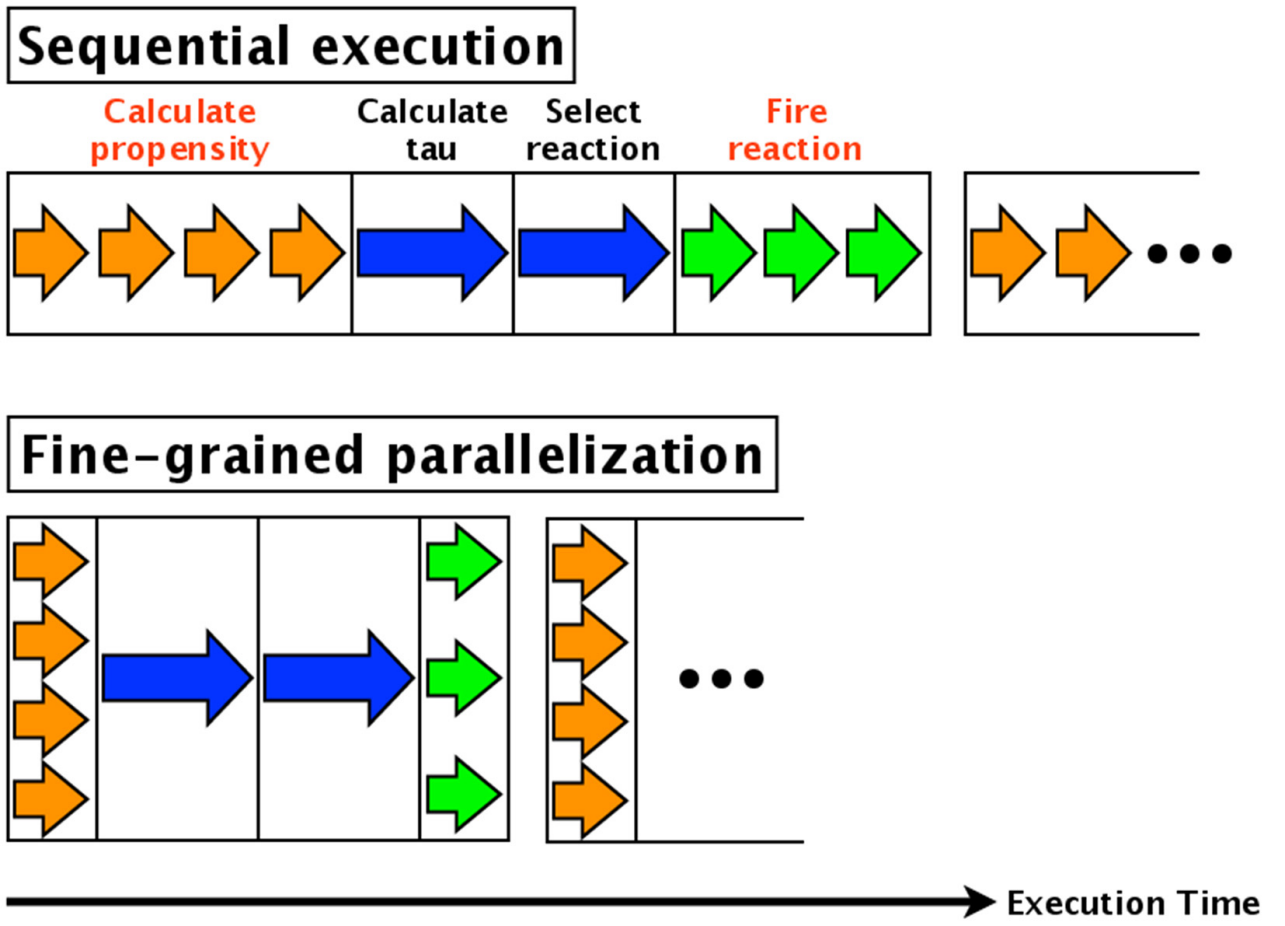
**Fine-grained parallelization of the direct method**. The blue arrow shows the calculation of the reaction time τ and the selection of a reaction; these cannot be parallelized. On the other hand, the orange arrow (calculation of propensity) and green arrow (updating the number of molecules) are independent processes that can be parallelized.

Fine-grained parallelization also parallelizes each component of each simulation. For example, in the direct method, the calculations of the propensities (step 3, Section 2.1) of the various reactions are parallelized. Similarly, updating the numbers of each molecular species (step 7, Section 2.1) is parallelized. The calculation time of step 3 is thus reduced by a factor equal to the number of reactions, and the time for step 7 is reduced by a factor equal to the number of molecular species affected. An overview of fine-grained parallelization of the direct method is shown in Figure 1; the arrows indicate the execution times of each step. The blue arrow in the figure indicates calculation of the reaction time τ and the selection of a reaction, and these cannot be parallelized. The orange and green arrows indicate calculation of the propensity and updating the number of molecules, respectively; these are independent processes and thus can be parallelized. As shown in Figure 1, the total execution time is reduced.

When implementing fine-grained parallelization with the CUDA programming language, we assigned a thread to the calculation of the propensity of each reaction. When updating the number of molecules, we assigned a thread to each molecular species.

### 2.4. Memory access optimization on a GPU

In the CUDA programming model, when a program is launched, data (e.g., matrices) are loaded from the host computer to the GPU's memory. The CPU on the host computer then sends a message to begin execution of the operation. Once the GPU has received this message, the arithmetic units begin to process in parallel as threads. Once all threads have been completed, the GPU returns the results to the host computer. Because multiple threads are executed simultaneously on a GPU, it is necessary to carefully design the access pattern of the threads in order to avoid collisions when they attempt to access the GPU memory to perform read or write operations.

There are various types of memory available in CUDA, including global, constant, texture, and shared; these differ in capacity and speed of access. Global memory has the largest capacity but requires the longest access time. To avoid the high latency of global memory, access to global memory should be coalesced (Nvidia, 2014). This means that all threads should follow a specific access pattern.

On the other hand, shared memory has a short access time, but its capacity is very limited. A benefit of using shared memory is that it has low latency. Shared memory has small capacity, so calculations must be partitioned (e.g., matrices and variables) and at any time, only the part being used is loaded to the shared memory. It is also important that access to shared memory be controlled in order to prevent collisions between threads. If there are 16 groups of physical addresses (banks), then the shared memory can give simultaneous access to 16 different threads. If multiple threads attempt to access the same bank, a “bank conflict” (Nvidia, 2014), this will result in sequential access, and thus result in high latency.

In our implementation, we stored the time course in the global memory and stored the number of molecules and propensities in shared memory. In this way, writing to the global memory was coalesced, and bank conflicts are avoided.

**Figure 2:**
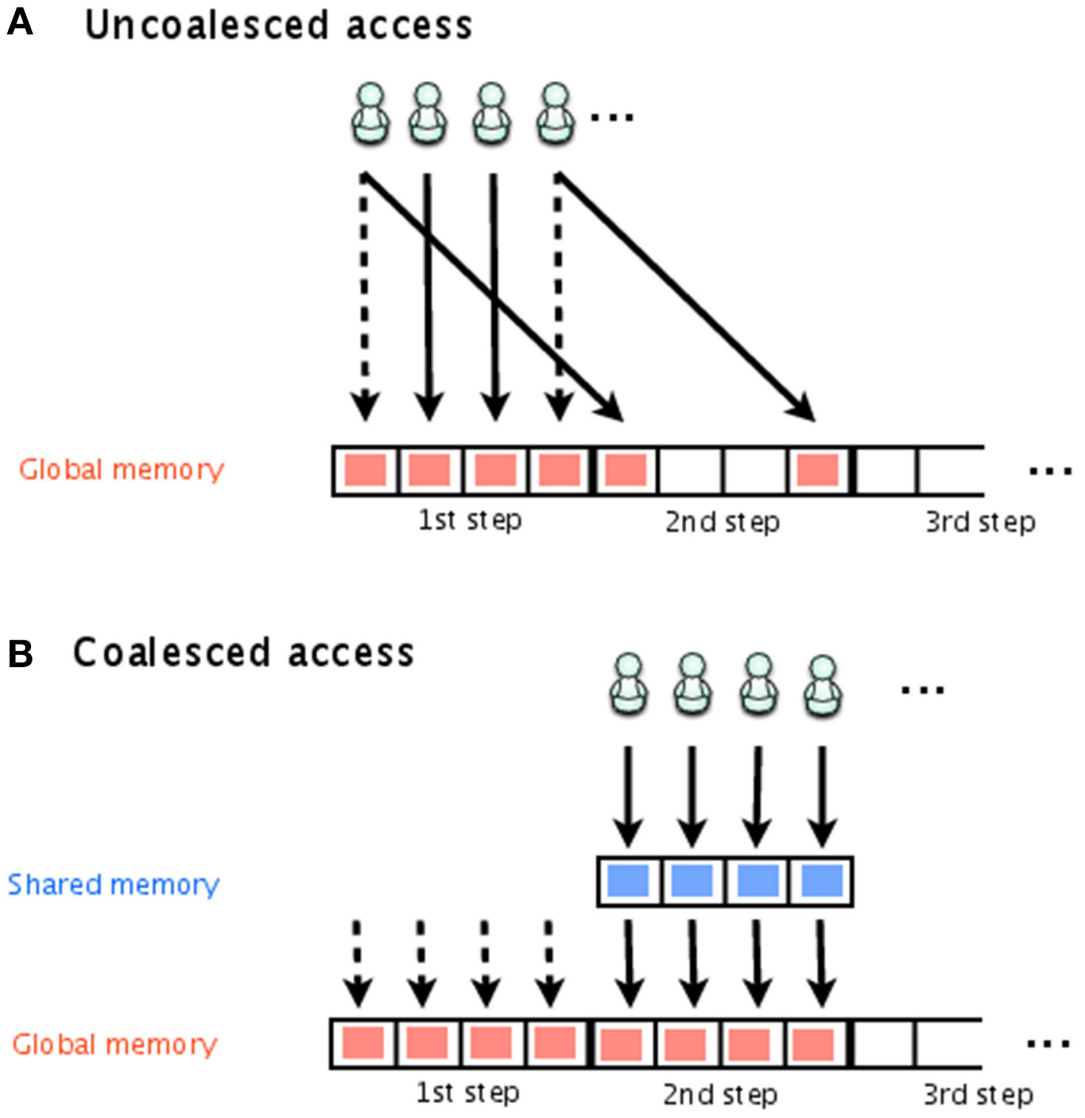
**Uncoalesced and coalesced access to global memory**. Stick figures indicate the threads that access global memory for the storage of the simulation results. In **(A)**, threads 1 and 4 have already finished storing their results for the first step and are attempting to store the results for the second step; threads 2 and 3 are attempting to store their results for the first step. This is an example of uncoalesced access. In **(B)**, shared memory is used to temporarily store the simulation results, which are later transferred together to global memory. This is an example of coalesced access.

Figure 2 shows storing of the time course, with both uncoalesced and coalesced access to global memory. Each thread is indicated by a stick figure. In this figure, it is assumed that there are four simulations executed simultaneously and that each one has its own thread for storing the results. Even though they are all simulating the same model, the execution time of each simulation will differ depending on one of the random numbers. In Figure 2A, threads 1 and 4 have already finished storing their results for the first step and are attempting to store their results for the second step, while threads 2 and 3 are attempting to store their results from the first step. This results in uncoalesced access. To avoid this problem, we temporarily store the results in shared memory, and then transfer the results from all threads to global memory at the same time, as shown in Figure 2B.

**Figure 3:**
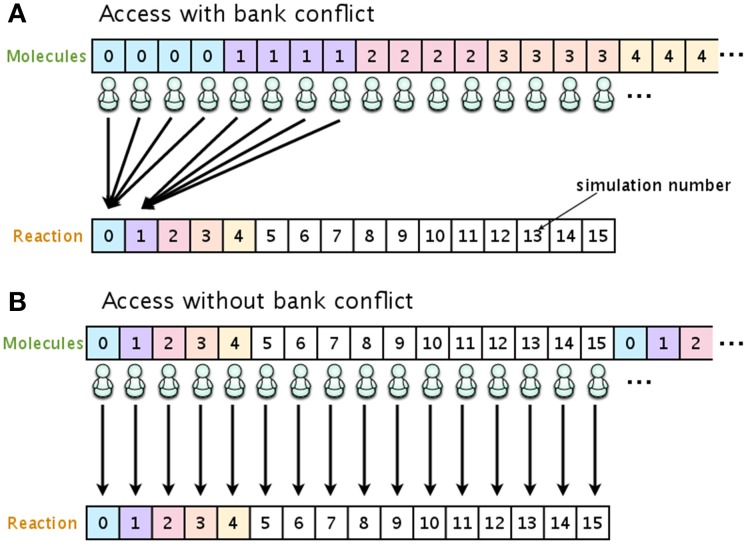
**Optimizing the location of data on the shared memory in order to avoid bank conflicts**. If the number of molecular species and reaction types are allocated as shown in **(A)**, multiple threads will have access to the same bank, and this will result in cause bank conflicts. Allocating the data as shown in **(B)** avoids bank conflicts.

To eliminate the risk of a bank conflict, we optimized the location of the data on the shared memory. In our implementation, the shared memory is used to store the number of molecules and the propensity functions. Figure 3 shows examples of access to shared memory with and without a bank conflict. In both Figures 3A,B, the upper arrays store the numbers of molecules in each species, and the lower arrays store the reaction type to be fired in the current simulation step. The number in each element of the array represents the simulation number (id). In this example, there are 16 simulations running simultaneously, and each simulation consists of four different molecular species. When it is time to update the number of molecules (step 7, Section 2.1), if the data are located as shown in Figure 3A, multiple threads will attempt to access the same bank (an element in the lower array), which will cause a bank conflict. To avoid this bank conflict, we have located the data as shown in Figure 3B. With this optimization, each element of the array is accessed by only a single thread, and thus bank conflicts are avoided.

### 2.5. Reduction of the time to transfer data between the GPU and the host computer

As described in Section 2.4, prior to executing a simulation, it is necessary to transfer data from the host computer to the GPU. Usually, the time required to do this is not negligible, and it adds to the total execution time. To estimate this overhead quantitatively, we have implemented a prototype of SSA on a GPU, and we profiled its execution time, as shown in Table 1. The most time-consuming task was found to be memory allocation, and this occupied almost 40% of the total execution time. The reason for this is that we store all of the time course results, which requires a large amount of memory. Data transfer is also time consuming, and it occupies 25% of the total execution time. When the time course results occupy a large amount of the GPU memory, the data transfer time from the GPU to the host computer will also increase. To overcome this problem, we implemented an asynchronous transfer scheme for moving data from the GPU to the host computer.

**Table 1:** **Execution time profile**.

**Procedure**	**% of total execution time**
Memory allocation (page lock)	38.0
Data transfer	25.0
Execution of kernel	23.3
Random number generation	13.3
Other	0.4

**Figure 4:**
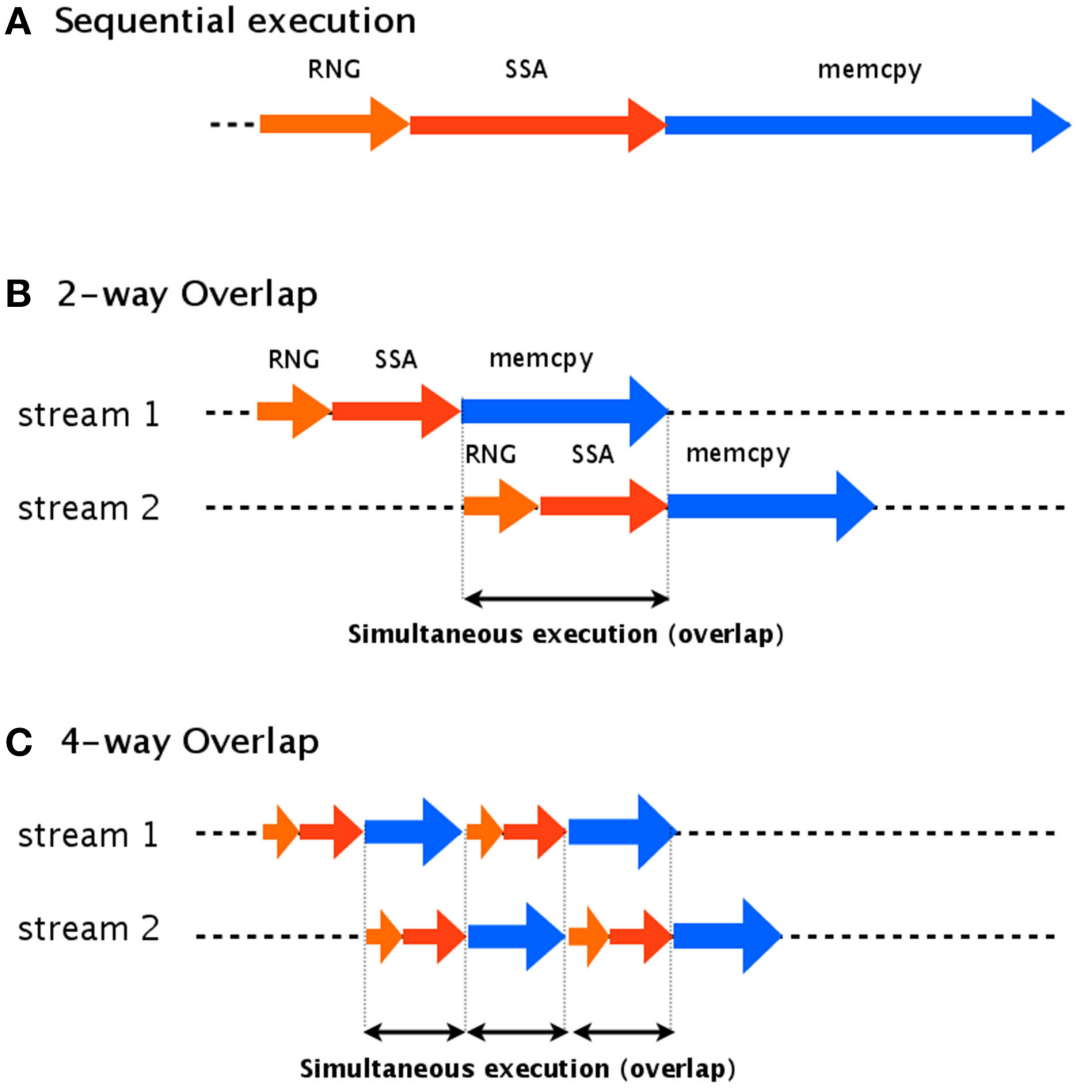
**Two-way and four-way overlap streaming of data transfer**. In an asynchronous transfer scheme, the data is split into multiple streams which are executed in parallel. **(A)** Shows an example of sequential execution, which has no overlap. **(B)** Shows an example of two-way overlap, which executes two streams: stream 2 is able to begin its computations while stream 1 is still transferring its results. In four-way overlap, the simulation task is split into four parts **(C)**. This results in an increase to three overlaps, compared to only one overlap in two-way overlap.

The idea of our asynchronous transfer scheme is to split the simulation into multiple streams and then execute these streams in parallel. Each stream contains random number generation (RNG), a stochastic simulation (SSA), and transfer of the data to the host computer (memcpy), as shown in Figure 4. If each stream runs independently, one thread can continue its computation on the GPU (kernel execution) while another stream is transferring the result to the host computer.

To implement this asynchronous data transfer scheme, we split the simulation tasks into two parts: kernel execution and data transfer. A schematic diagram of asynchronous transfer with two streams is shown in Figure 4B. While one stream (stream 1) is transferring its results to the host computer (memcpy), another (stream 2) begins to execute its kernel. This transfer scheme is called a two-way overlap. Note that in two-way overlapped data transfer, the data transfer time of stream 1 is suppressed by the kernel execution time of stream 2. Under ideal conditions, the data transfer time will be cut in half. In our implementation, we applied four-way overlapped data transfer. The difference between two-way and four-way overlap is the number of parts into which the stream is split. In four-way overlapped data transfer, the simulation is split into four parts, as shown in Figure 4C; this results in three areas of overlap, compared to only one for two-way overlap. Under ideal conditions, the data transfer time will be cut by a factor of four.

### 2.6. Data compression

Since CUDA requires that all of the data be loaded onto the GPU memory, the capacity of the GPU memory is a bottleneck. Unfortunately, it is impossible to extend the size of the memory of a GPU, although extending the memory is straightforward and cost effective on general-purpose computers. Moreover, the memory of a GPU is usually less than that of a personal computer. For example, the NVIDIA Tesla C1060, which we used for this study, has 4 GB of memory, while most desktop computers used for scientific calculations have more than 8 GB of memory, and, as mentioned, it is easy to increase the memory. Acceleration of processes on a GPU always encounters this problem; thus, effectively reducing the memory footprint is another important issue for such implementations.

In our implementation, we used the global memory to store the time course results and the constant memory to store the reaction rate constants and the stoichiometry matrix. The constant memory has low latency and small capacity (64 KB), compared with the global memory, and it is read-only access. Because the stoichiometry matrix and reaction rate constants do not change during the simulation, we located them in the constant memory. The structure that consumes the most memory is the stoichiometry matrix used in the SSA; however, this matrix is usually sparse, and so we implemented compressed row storage (CRS) to reduce its footprint.

**Figure 5:**
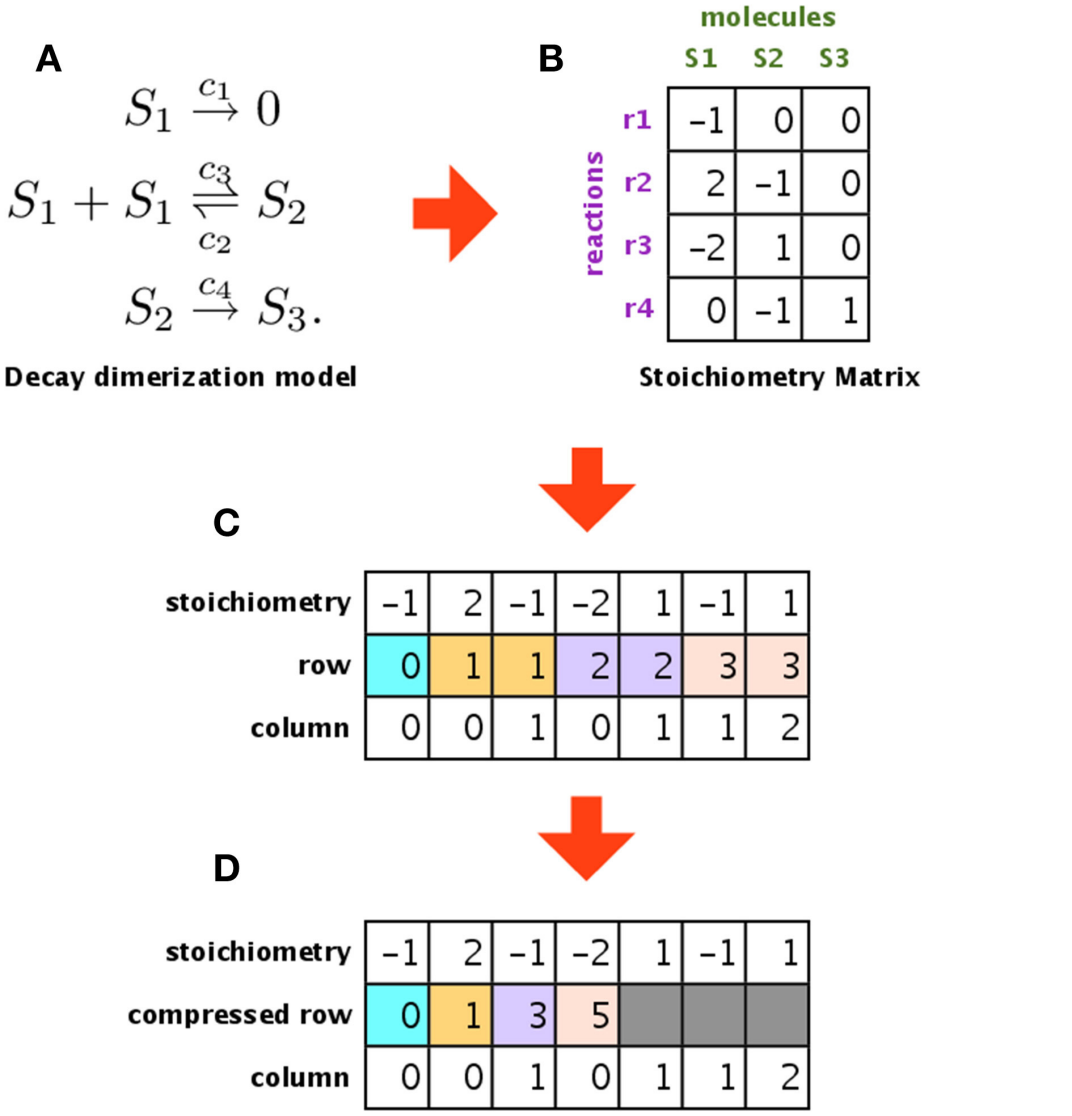
**Compressing the stoichiometry matrix using compressed row storage**. **(A,B)** Show a decay dimerization model and its stoichiometry matrix, respectively. Each row of the stoichiometry matrix represents a molecular species that will be synthesized or destroyed by one of the reactions. **(C)** Shows a matrix that only stores the non-zero entries in the stoichiometry matrix, along with the original row and column indices. **(D)** Is a compressed matrix of **(C)**, obtained by eliminating duplicates of the same index in the second row.

Figure 5 shows an example of a model, its stoichiometry matrix, and the compressed matrix. Figure 5A shows an example of a biochemical system (decay dimerization model). It consists of three molecular species (*S*_1_–*S*_3_) and four reactions (*r*_1_–*r*_4_). This biochemical system can be represented by a stoichiometry matrix, as shown in Figure 5B. Each row of the stoichiometry matrix represents a molecular species that is synthesized or degraded by one of the reactions. If the value is zero, then the corresponding molecular species is not included in the reaction for that column (in other words, the simulator does not have to consider this molecular species for this reaction). Most biochemical reaction networks are loosely coupled, and so the stoichiometry matrix is sparse Li and Petzold (2010). To compress this sparse matrix, we extracted the non-zero values and generated a new matrix that contains only these non-zero values and their original row and column indices, as shown in Figure 5C. This new matrix still has some redundant information, in that the row indices are repeated (e.g., 1, 1, 2, 2, 3, 3). We used CRS to avoid this redundancy and to store only the column indices in each compressed row, as shown in Figure 5D. By using CRS to convert the stoichiometry matrix, we succeeded in storing a decay dimerization model that had approximately 1400 reactions; for the same amount of memory, an unconverted matrix could only store approximately 120 reactions.

### 2.7. Importing the model

Although not all studies of simulations on GPUs have mentioned or satisfied this requirement, it is crucial to separate the model from the implementation in order to provide a convenient software tool. If the model is hard-coded in the simulator, the program must be rewritten whenever the model is modified; the entire code must then be recompiled. This causes a problem for those end users who are not familiar with the necessary development tools. In particular, this can cause a high barrier for GPGPU, since most end users are not proficient in GPU programming.

To avoid this problem, we designed our simulator so that the model is imported; thus, our software package can be distributed in binary and does not need to be compiled by the end user. Our system uses the Systems Biology Markup Language (SBML), which is a tool-neutral computer-readable format for representing models of biochemical reaction networks; it is applicable to metabolic networks, cell signaling pathways, gene regulatory networks, and other modeling problems in systems biology (Hucka et al., 2003, 2004; Podlozhnyuk, 2007). To import SBML, we use LibSBML (Bornstein et al., 2008) to easily access the SBML elements from the C programming language. The host computer converts the SBML elements (such as reactions, molecular species, and rate constants) to matrices, and then loads them into the GPU memory. Once the matrices have been successfully loaded, the simulator launches a kernel to start the simulation. All of the sample models that were used for evaluation of this procedure were described using SBML.

## 3. Results

In this section, we will evaluate our implementation. For comparison, we implemented the direct method in the C programming language for sequential execution on a CPU. We compared the execution time of a stochastic simulation of the same model performed on both a CPU and a GPU. The GPU we used was an NVIDIA Tesla C1060, mounted on a host computer that had Core i7 2.80 GHz with 12 GB of memory. The CPU version of our simulator was executed on the host computer. The model we chose for the benchmark was a decay dimerization model, which consisted of four reactions and three molecular species, as follows:

(1) $$S_{1}\overset{c_{1}}{\to }0$$

(2) $$S_{1}+S_{1}\underset{c_{2}}{\overset{c_{3}}{⇌}}S_{2}$$

(3) $$S_{2}\overset{c_{4}}{\to }S_{3}$$

This model is quite simple, but it is known to cause stochastic fluctuations, and a similar reaction system appears in previous research by McAdams and Arkin (1997). The decay dimerization model was also used as a benchmark model by Li and Petzold (2010), and we applied the same simulation conditions as used in that study; these conditions are shown in Table 2.

**Table 2:** **Simulation conditions for the decay dimerization model**.

**REACTION RATE CONSTANTS**	
*c*_1_	1.0
*c*_2_	0.002
*c*_3_	0.5
*c*_4_	0.04
**INITIAL CONDITIONS**	
*S*_1_	10,000
*S*_2_	0
*S*_3_	0
Simulation steps	11,000

### 3.1. Hybrid parallelization

**Figure 6:**
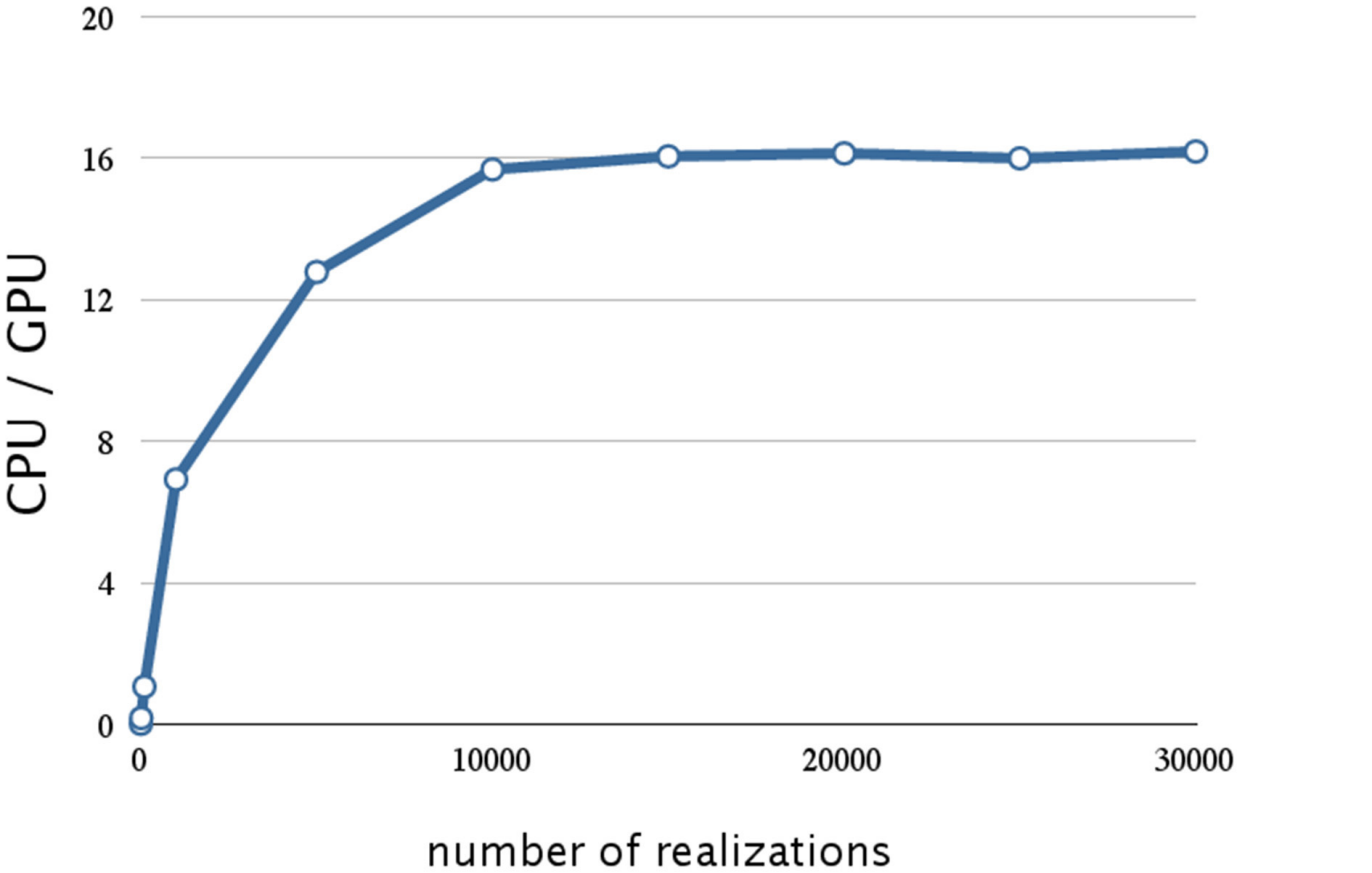
**Performance analysis of hybrid parallelization w.r.t. the number of realizations**. This figure shows a plot of the ratios of the execution times for the decay dimerization model (Table 2) on a CPU(Core i7 2.80 GHz with 12 GB of memory) and on a GPU (NVIDIA Tesla C1060) as the vertical axis, and the number of realizations as the horizontal axis. With hybrid parallelization and for greater than 1000 realizations, the process is up to 16 times faster than on a CPU.

We evaluated the effect on SSA of hybrid parallelization, which is a combination of fine-grained and coarse-grained parallelization. Hybrid parallelization simultaneously executes multiple stochastic simulations as coarse-grained parallelization, and simultaneously calculates the propensity functions and updates the number of molecules for each stochastic simulation as a fine-grained parallelization. The execution time of the direct method with different numbers of realizations is shown in Table 3, and the ratio of the execution time on a CPU to that on a GPU is shown in Figure 6. From Table 3 and Figure 6, we can see that there is no performance gain on a GPU when the number of realizations is small (<100), but if the number of realizations is large (>1000), the effect is apparent. We found that hybrid parallelization was up to 16 times faster than implementation on a CPU.

**Table 3:** **Execution times with different numbers of realizations**.

**Number of realizations**	**Execution time (s)**		**CPU/GPU**
	**CPU**	**GPU**	
1	0.001	0.079	0.01
10	0.015	0.086	0.17
100	0.17	0.160	1.06
1000	1.91	0.276	6.92
5000	14.01	1.096	12.78
10,000	35.05	2.235	15.68
15,000	52.57	3.275	16.05
20,000	70.10	4.344	16.14
25,000	87.82	5.490	16.00
30,000	105.15	6.495	16.19

### 3.2. Memory access optimization

**Figure 7:**
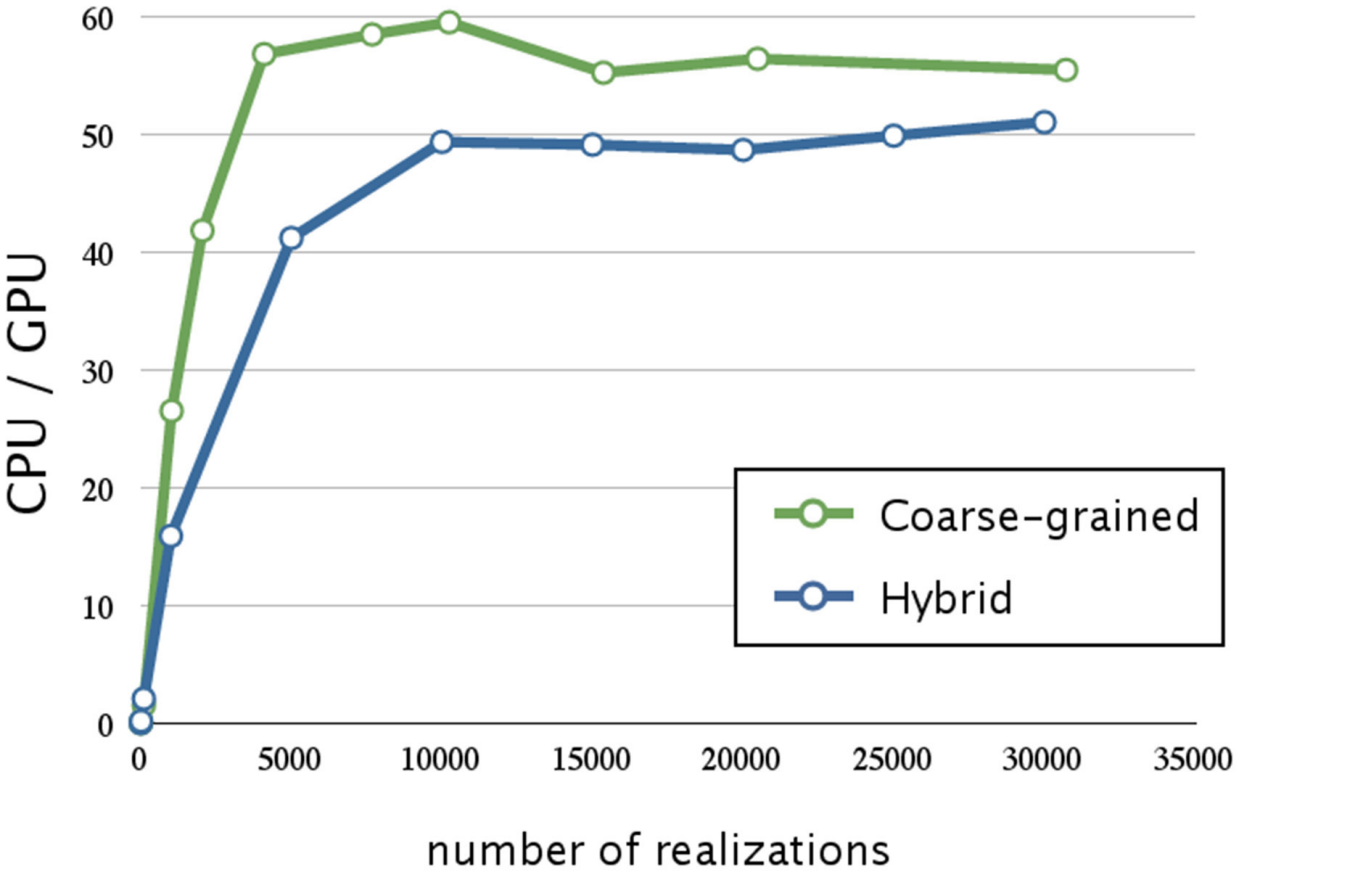
**Performance analysis of memory access optimization w.r.t. the number of realizations**. This figure shows a plot of the ratios of the execution times for the decay dimerization model (Table 2) on a CPU (Core i7 2.80 GHz with 12 GB of memory) and on a GPU (NVIDIA Tesla C1060) as the vertical axis, and the number of realizations as the horizontal axis. The blue and green lines represent the acceleration obtained by memory access optimization with hybrid and coarse-grained parallelization, respectively. Applying memory access optimization improved performance by a factor of 3.1; this was 50 times faster than on a CPU. Memory access optimization with coarse-grained parallelization achieved an even greater improvement than that of hybrid parallelization (it was 60 times faster than on a CPU).

Next, we evaluated the effect of optimizing the memory access. The ratios of execution times on a CPU and that on a GPU are shown in Figure 7; the blue line indicates the acceleration obtained by optimizing the memory access and using hybrid parallelization. Optimizing the memory access resulted in improving the time by a factor of 3.1; the overall result was 50 times faster than that on a CPU. In Figure 6, we see that there is less improvement when the number of realizations is small, because the parallelization has a smaller effect. On the other hand, optimizing the memory access on a GPU greatly improved performance when there was a large number of realizations. This result suggests that the memory access on a GPU is a bottleneck; thus, it is essential to profile the access pattern of the code and optimize the data location and structure.

The green line in Figure 7 indicates the acceleration obtained by optimizing the memory access and using coarse-grained parallelization. Interestingly, optimizing the memory access had a greater impact when using coarse-grained parallelization than when using hybrid parallelization; its execution was 60 times faster than on a CPU. This may be because coarse-grained parallelization requires less synchronization between the threads than does hybrid parallelization, and thus the threads may be executed more efficiently. We also note that coarse-grained parallelization requires relatively simple memory access compared to that required by hybrid parallelization, and this is advantageous. Although hybrid parallelization may have the greatest advantage for huge models with very large numbers of reactions, we decided to implement other acceleration methods for use with coarse-grained parallelization.

### 3.3. Reduction of data transfer time

**Figure 8:**
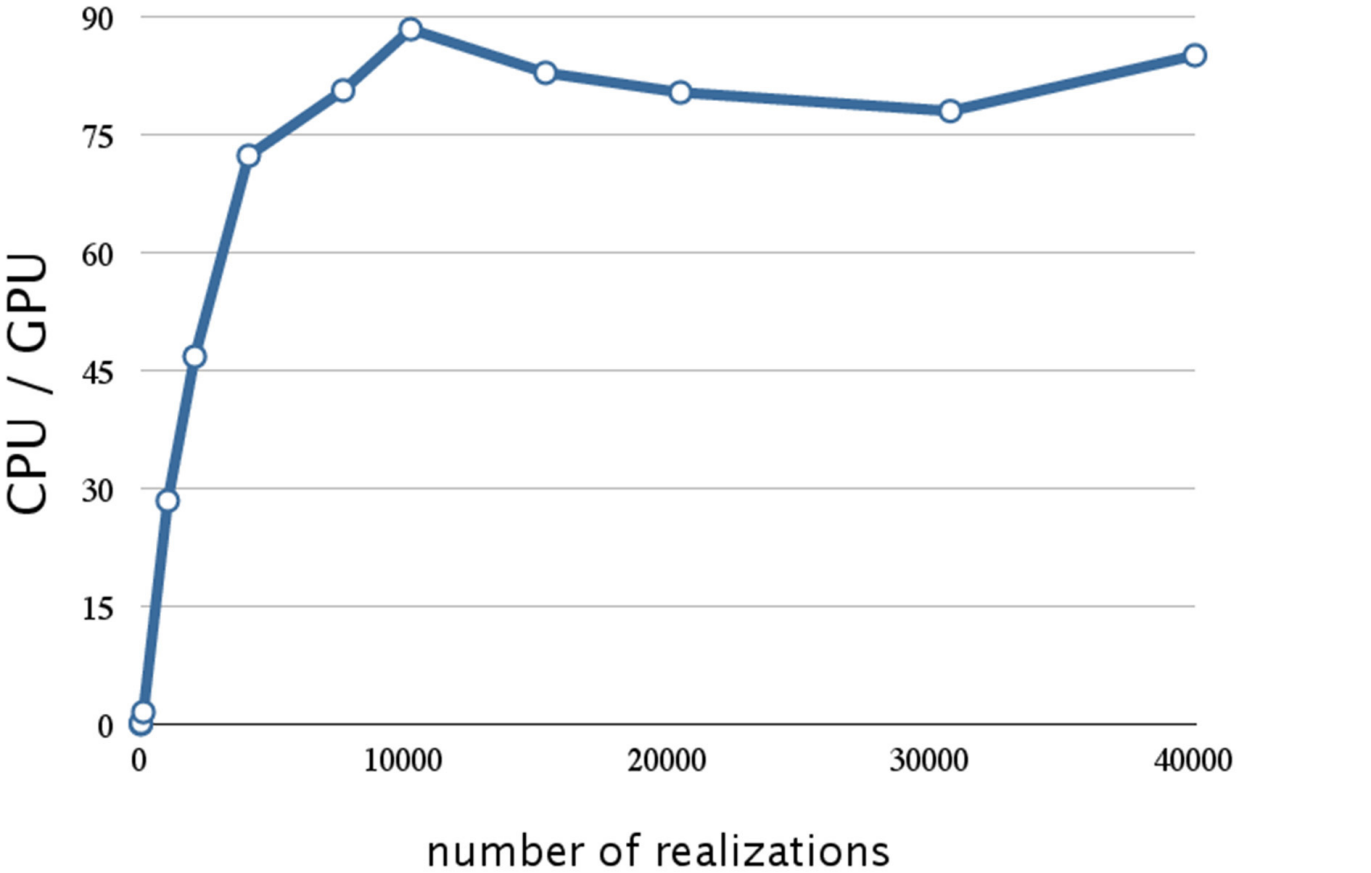
**Analysis of the reduction of the data transfer time achieved by four-way overlap w.r.t. number of realizations**. This figure shows a plot of the ratios of the execution times for the decay dimerization model (Table 2) on a CPU (Core i7 2.80 GHz with 12 GB of memory) and on a GPU (NVIDIA Tesla C1060) as the vertical axis, and the number of realizations as the horizontal axis. Applying the asynchronous data transfer scheme resulted in a further improvement by a factor of 1.5; the result was about 90 times faster than on a CPU.

In addition to parallelization and memory access optimization, we evaluated the improvement in performance achieved by the reduction of the time to transfer data. The execution times of 10,240 realizations with different methods of optimizing the data transfer (*n*-way overlap) is shown in Table 4, and Figure 8 shows the ratio of the execution times on a CPU and a GPU, with four-way overlapped data transfer. Note that the total execution time when there was no overlap (0.67 s; Table 4) is about one third that for 10,000 realizations (2.235 s; Table 3). This is due to the optimization of memory access, as described in Section 3.2.

**Table 4:** **Execution times for 10,240 realizations with different methods of optimizing data transfer**.

	**Kernel (s)**	**Data transfer (s)**	**Total execution time (s)**
No overlap	0.22	0.15	0.67
Two-way overlap	0.22	0.08	0.59
Four-way overlap	0.22	0.04	0.45

From Figure 8, we see that this implementation has a further improvement by a factor of 1.5 (this is about 90 times faster than on a CPU). The reduction in the data transfer time was motivated by noting that this consumed 25% of the execution time in our prototype implementation, as shown in Table 1. By implementing an asynchronous data transfer scheme, we reduced the data transfer time, to 50% and 25% of the original time for two-way and four-way overlap, respectively (see Table 4); this resulted in an improvement in performance by a factor of 1.5.

### 3.4. Data compression

As described in Section 2.6, using CRS to compress the stoichiometry matrix markedly reduced the memory footprint of the GPU implementation. To analyze the effect of this on the execution time, we created some sample models of various sizes. Each sample model consisted of several units of a single-gene production-reduction submodel. As an example, such a submodel consisting of two molecular species and two reactions is as follows:

(4) $$G\overset{c_{5}}{\to }G+P$$

(5) $$P\overset{c_{6}}{\to }0$$

For example, if a model consists of four independent single-gene production-reduction submodels, the model will contain eight molecular species and eight reactions. Because only one or two molecular species are involved in each reaction in each submodel, the stoichiometry matrix of the combined model will be sparse. Thus, we can expect that the use of CRS will have a notable effect. We created six models with different numbers of reactions (in the range of 8–256).

**Figure 9:**
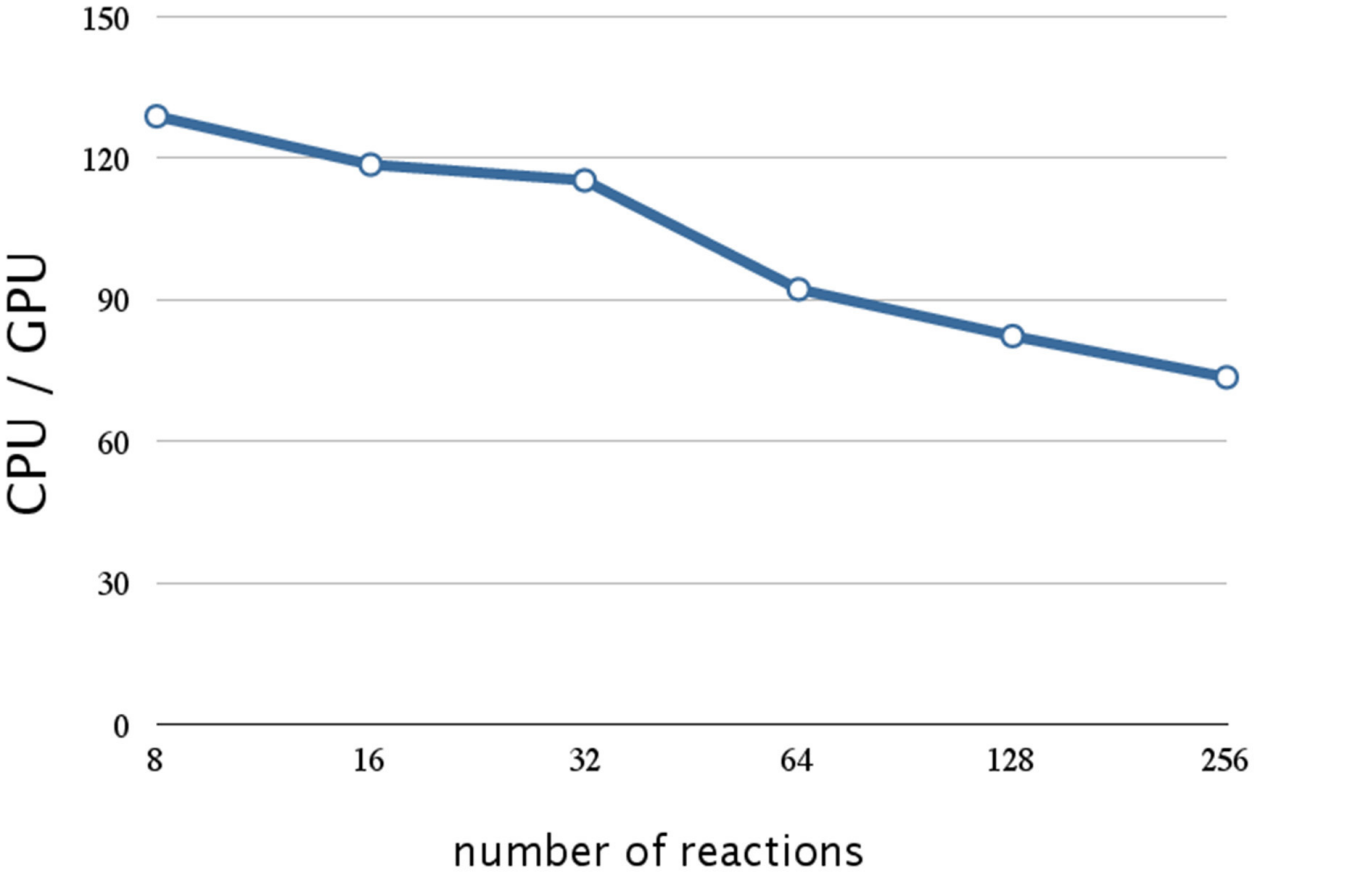
**Performance analysis of data compression w.r.t. the number of reactions**. This figure shows a plot of the ratios of the execution times of 10,240 realization of a single-gene production-reduction model on a CPU (Core i7 2.80 GHz with 12 GB of memory) and on a GPU (NVIDIA Tesla C1060) as the vertical axis, and the number of reactions (model size) as the horizontal axis. Compression of the stoichiometry matrix resulted in a further improvement by a factor of 1.4 for a model with eight reactions; this was about 130 times faster than on a CPU. On the other hand, the execution time on a GPU was notably longer for larger models (>64 reactions).

The execution times of 10,240 realizations with the different sizes of model are shown in Table 5, and the ratios of the execution times on a CPU and a GPU are shown in Figure 9. As a result of compressing the stoichiometry matrix, the stochastic simulation for a model with eight reactions is about 130 times faster on a GPU than on a CPU. This improvement was due to the implementation of CRS, and it was because of the sparseness of the data. In the previous implementation, it was necessary to perform a two-dimensional scan of the stoichiometry matrix in order to determine which molecules should be updated; with the CRS, the molecular information is stored as an index; thus, it is not necessary to scan the matrix, and the number of molecules can be updated with a minimal computational cost (Figure 5).

**Table 5:** **Execution times of 10,240 realizations with various sizes of model**.

**Number of reactions (model size)**	**Execution time (s)**		**CPU/GPU**
	**CPU**	**GPU**	
8	58	0.45	128.89
16	70	0.59	118.64
32	98	0.85	115.29
64	142	1.54	92.21
128	237	2.88	82.29
256	406	5.52	73.55

Although this implementation resulted in a drastic improvement in performance, the execution time on a GPU was notably longer for larger models (>64 reactions), as shown in Figure 9. This is not caused by the firing of the reaction (step 7, Section 2.1), but by the calculation of the propensity function and the selection of the reaction (steps 3, 4, and 6 Section 2.1); this is because the execution time of these procedures increases with an increase in the number of reactions.

We now consider the effect of CRS on the memory footprint of the stoichiometry matrix. Assume that a model consists of *r* reactions and *m* molecular species; the size of the stoichiometry matrix *S_sm_* will be

(6) $$S_{sm}=m\;\times \;r$$

and after using CRS, the size of the new stoichiometry matrix *S_crs_* will be

(7) $$S_{crs}=r\;+\;2\alpha$$

where α is the total number of elements in the CRS. Assuming *m* molecules are involved, on average, as reactants or products of each reaction, α will satisfy α = *m* · *r*; thus the size of the modified matrix is as follows:

(8) $$S_{crs}=(2\bar{m}\;+\;1)\times r$$

From Equations (6, 8), the difference in the memory footprint depends on the values of *m* and *m*. In the direct method (step 3, Section 2.1), *m* will be a value between zero and two; CRS will result in a smaller memory footprint even with a small model.

## 4. Discussion

In this section, we will summarize and discuss the results of our implementation.

Table 6 summarizes the acceleration methods implemented in this work and the ratios of the execution times compared with the implementation of the direct method on a CPU (CPU/GPU). From Table 6, it can be seen that the memory access optimization resulted in the greatest improvement in the performance, followed by the asynchronous data transfer and data compression. Although a GPU has the potential to be used for high-performance computing, its computational power cannot be harnessed by simply parallelizing an algorithm; this is because the way in which data is accessed during execution is a critical factor in GPU computing. We demonstrated this with our results in Section 3.2.

**Table 6:** **Summary of accelerated stochastic simulator and its various acceleration methods**.

**Methods**	**Parallelization algorithm**	**Acceleration (CPU/GPU)**
Parallelization of the direct method	Hybrid	16
Memory access optimization	Hybrid	50
	Coarse-grained	60
Asynchronous data transfer	Coarse-grained	90
Data compression	Coarse-grained	130

The primary feature that differentiates a discrete stochastic simulation from a continuous simulation (such as numerical integration of a differential equation) is the use of random numbers. When numerically integrating a differential equation, it is obvious that, given identical initial conditions, each simulation will produce identical results. On the other hand, stochastic simulations require multiple realizations, the results of each one being determined by random numbers. Parallelization on a GPU is well-suited for this kind of simulation, because a simulator can share one model for multiple realizations, which reduces the memory footprint. Choosing the best parallelization algorithm from fine-grained, coarse-grained, and hybrid parallelization is another important need with GPU computing. In principle, coarse-grained parallelization is the most efficient method for multiple realizations, because it only requires infrequent synchronization between the threads. For the problem that we considered in this study, multiple realizations using one model was a requirement, so parallelizing one realization (fine-grained parallelization) was not as effective as parallelizing multiple realizations (coarse-grained parallelization), because of the need for frequent synchronization. This was also shown in Section 3.2. Fine-grained parallelization has the potential to accelerate a simulation when an objective model contains a large number of reactions and/or a large number of molecular species, which result in high computational cost for the calculation of the propensity functions. Although we did not consider fine-grained parallelization after Section 3.2, preliminary results were shown in Section 3.4. It was shown that the performance improvement obtained by coarse-grained parallelization will decrease logarithmically with the model size, as shown in Figure 9. This result suggests that it might be possible to solve this problem by calculating the propensity function using fine-grained parallelization. The efficiency of parallelization can be measured by the occupancy', which is defined to be the number of active thread groups divided by the maximum number of thread groups. If there is a synchronization between threads during a simulation, some preceding threads will be required to wait until the remaining threads reach the synchronization point. The number of waiting threads will decrease the occupancy, because they will be included in the denominator. The occupancy depends on the particular problem and the parallelization method, but in principle, hybrid parallelization can lead to lower occupancy than that of coarse-grained parallelization. Applying hybrid parallelization is challenging, since high occupancy must be maintained.

Functionality for storing all of the time course data during a realization is an essential feature for understanding the dynamics of a model. We note that this functionality is found in most existing software tools that support stochastic simulation (Ramsey et al., 2005; Hoops et al., 2006; Mauch and Stalzer, 2011; Sanft et al., 2011). An existing proposal for the acceleration of the direct method using a GPU (Li and Petzold, 2010) performs faster than our method (speedups by a factor of about 200), but it lacks the functionality for storing all the time course data, which not only consumes memory but also increases execution time. Our intent was to add functionality to store the time course data while improving performance. We used an asynchronous data transfer scheme so that the time course data would be transferred during the simulation and thus decrease the data transfer time (Figure 8). Overall, we achieved a speedup by a factor of 130 compared with a sequential realization on a CPU.

Our evaluation was performed on an NVIDIA Tesla C1060, which has 240 arithmetic units (cores) and 4 GB of memory. The peak performance of the C1060 is 933 Gflops in single-precision floating point format. Several GPUs have been released by NVIDIA for the purpose of GPGPU. For example, the NVIDIA Tesla K40, which is a high-end product with 2880 cores and 12 GB of memory, provides 4.29 Tflops at peak single-precision floating-point performance. By implementing our method on a high-end GPU, we would expect a greater improvement in performance. The performance is not affected only by the number of flops; current GPUs have a higher compute capability (3.5) compared with the C1060 (1.3). The difference in compute capability directly affects the memory access performance. In principle, higher compute capability will place a lower penalty on uncoalesced access and looser restrictions on coalesced access and bank conflicts. Benchmarking on a GPU with higher compute capability might show different results for improvements when using different acceleration methods.

In this study, we applied parallelization and several acceleration methods to the direct method, which is the most straightforward way to implement the SSA of Gillespie. As described in Section 2.1, there are several algorithms for the SSA, and the use of simulation algorithms can improve the total throughput. The next targets for improved implementation are the optimized direct method (Cao et al., 2004) and the sorting direct method (McCollum et al., 2006). The optimized direct method optimizes the order of $\sum_{i\;=\;0}^{n\;-\;1}a_{i}$ (in step 6, Section 2.1) to reduce the calculation time. The sorting direct method is another improvement of the direct method. The difference between the optimized direct method and the sorting direct method is a pre-simulation step, in which the optimized direct method sorts the propensity functions. Since both algorithms are based on the direct method, extending our implementation to them is expected to have a notable effect on parallelization.

In this work, we have designed and implemented several parallelization algorithms and acceleration methods for the SSA. We have included a time course recording function while accelerating SSA simulations by a factor of up to 130. GPUs are known to be a cost-effective and power-saving solution for high-performance computing. With the added functionality for recording the time course and the ability to import a model that is described in SBML, we hope that our implementation will contribute to the field of systems biology, in which modeling and simulation play important roles in understanding complex biological systems.

## Author contributions

AF conceived of the study and coordinated the project. KS designed and implemented the majority of the source code; KH and AF participated in the design of the acceleration scheme. NH and AF supervised the project. KS, NH, and AF wrote the manuscript. All authors read and approved the final manuscript.

## Funding

This work was supported by JSPS KAKENHI Grant Numbers 23136513 and 24300112.

### Conflict of interest statement

The authors declare that the research was conducted in the absence of any commercial or financial relationships that could be construed as a potential conflict of interest.
